# Biogenic silica-based microparticles obtained as a sub-product of the nanocellulose extraction process from pineapple peels

**DOI:** 10.1038/s41598-018-28444-4

**Published:** 2018-07-10

**Authors:** Yendry R. Corrales-Ureña, Carlos Villalobos-Bermúdez, Reinaldo Pereira, Melissa Camacho, Eugenia Estrada, Orlando Argüello-Miranda, Jose R. Vega-Baudrit

**Affiliations:** 1 National Laboratory of Nanotechnology LANOTEC - National Center of High Technology CeNAT, 1.3 km north from the USA embassy, San José, Costa Rica; 2 Technical University of Costa Rica, UTN, San Carlos, Costa Rica; 3 National University of Costa Rica, UNA, Heredia, Costa Rica

## Abstract

Silica in plant tissues has been suggested as a component for enhancing mechanical properties, and as a physical barrier. Pineapples present in their shell and bracts rosette-like microparticles that could be associated to biogenic silica. In this study, we show for the first time that silica-based microparticles are co-purified during the extraction process of nanocellulose from pineapple (*Ananas comosus*). This shows that vegetable biomass could be an underappreciated source, not only for nanocellulose, but also for a highly valuable sub-product, like 10 µm biogenic rosette-like silica-based microparticles. The recovery yield obtained was 7.2 wt.%; based on the dried initial solid. Due to their size and morphology, the microparticles have potential applications as reinforcement in adhesives, polymer composites, in the biomedical field, and even as a source of silica for fertilizers.

## Introduction

Biomass derived from relevant agricultural crops such as coffee, bananas, sugar cane and pineapple is usually discarded as waste, which is burned or in many cases accumulated in nearby forests. This environmental problem is evident in the world’s largest pineapple producer: Costa Rica; as well as other countries where the pineapple juice industry discards 50% to 65% of the residual biomass^1–3^. That represents an estimated 1.5 million tons of stubble per year^1^. The disposal of these residues is also a health concern because of disease transmitting mosquitos, the pest fly *Stomoxys calcitrans* and pathogenic fungi thrive in the moistly and sugary residues^4,5^. To mitigate this problem, alternative uses for the residual biomass have been proposed. Previously, pineapple peels were used mainly as a source of nanocellulose^6^. Interestingly, Ferreira *et al*. compared the morphoanatomy and ontogeny of six species of Bromeliaceae fruits from Brazil and showed that “silica bodies” are present in the longitudinally elongated cells forming the exocarp^7^. In this study, we focused on the isolation of such silica microparticles from pineapple peels.

The artificial production of sphere-like nano- and microparticles is achieved using silanes as raw material and organic solvents^8,9^, for instance, the hydrolysis and condensation of tetraethyl orthosilicate, TEOS (Si(OC_2_H_5_)_4_), in anhydrous ethanol followed by hydrolyzation in distilled water under basic condition^10^. These processes are expensive and energy demanding^8^. In contrast, the production of silica particles from residual biomass from crops such as rice is an economically and environmentally feasible option^11^. Alshatwi *et al*. pretreated rice husk residues with hydrochloric acid obtaining cellulose, hemicellulose and lignin while the remaining material was calcined to produce disk-like silica nanoparticles^12^.

In plants, silica is one of the stiffest cell wall components which provides mechanical strength and rigidity^13,14^. It is a physical barrier, protecting plants from multiple abiotic and biotic stresses, helping to improve heat tolerance and deterring herbivores^15–17^. The mechanism of biogenic silica production has been associated to the polymerization of Si(OH)_4_ and [SiO_x_(OH)_4–2x_]_n_ compounds which the plant obtains from soil^15^. The uptake kinetics is species specific. Silica cells are responsible for condensing silicic acid into silica by a physiologically regulated process. Silicon could be deposited as silica within and between cells and tissues, creating amorphous structures called phytoliths or silica bodies^14^. Silica is found in the cell wall matrix, primarily in outer epidermal walls, and within the lumen of some types of epidermal cells. Silicified structures are found in plants like rice, bamboo, sugarcane and salt grass species, among others^15,17^. In *Equisetum spp*, the epidermal surface has discrete knobs, rosettes and fibrillary/globular/sheet-like silica bodies^13^. For most plant species, however, the association of the silica particles with the cell-wall components is an ill-defined process^15^.

In this study, the location and characterization of the silicon dioxide-based bodies on the different parts of the pineapple fruit is reported. The extraction and yield recovery of biogenic rosette-like silica-based microparticles (BRSM) as a sub-product of the nanocellulose (NCC) extraction process from the pineapple peels is reported for first time. Techniques such as transmission electron microscopy (TEM), Fourier-transform infrared spectroscopy (FTIR), scanning electron microscopy (SEM), energy-dispersive X-ray spectroscopy (EDX), X-Ray diffraction (XRD), optical microscopy and thermogravimetric analysis (TGA) were used to characterize the materials.

## Methods

### Materials

The pineapple peels were obtained from different sources: a waste processing plant located in Muelle, San Carlos, from supermarkets and street fruit markets in San José, Costa Rica. Sulfuric acid (H_2_SO_4_), sodium hydroxide (NaOH), chloridric acid (HCl), Avicel PH-101, and sodium hypochlorite (NaClO) reagents were purchased from Sigma Aldrich. The multiwalled carbon nanotubes were characterized as reported before by the authors^18^ and the silica used as control was purchased from Merck (particle size 0.2–0.5 mm; chromatography).

### Nanocellulose and silica extraction process

The nanocellulose extraction process from pineapple peels was previously described by the authors, and a diagram of the process is shown in Supplementary Fig. S1. Briefly, the ground pineapple peels were incubated in NaOH to remove the lignin and hemicellulose, then bleached in NaClO, before incubating in HCl to hydrolyze the cellulose and to obtain microcellulose^6^. Between each step, the product was thoroughly rinsed with water until neutral pH, and the solid was recovered by centrifugation at 13000 rpm. In the final step, the material was hydrolyzed with H_2_SO_4_ to obtain NCC. Two fractions of particles were separated from this acid solution by centrifugation at 2500 rpm; the supernatant containing mainly particles in the nanometer range and the precipitate containing the micrometer solid fraction and insoluble materials. The precipitate was mainly BRMS. To purify the BRMS of cutin and NCC residues as well as remove the carbon-based materials surrounding the particles, the solid was incubated with a second H_2_SO_4_ solution. Sulfuric acid solutions of 65, 30, 15 and 2 wt.% were tested between 30 minutes and 4 hours at 55 °C (1 g of precipitate/10 ml of H_2_SO_4_ solution) to determine the lowest acid concentration needed to disperse the carbon derivatives in the solution. The second H_2_SO_4_ acid step was applied to partially depolymerize the cutin bound to polysaccharides^19^. Finally, the product was separated from the liquid viscous phase by centrifugation at 2500 rpm and rinsed several times with deionized water. For FTIR analysis, the cutin derivatives were separated from NCC by phase separation at 13000 rpm.

### Structural analysis

#### Optical microscopy

The peel sections were cut using a new sharp knife and without any treatment on a light microscope (Motic BA 410), maintaining the tissue wet during analysis.

#### Scanning electron microscopy (SEM) and energy dispersive analysis (EDX)

The samples were analyzed using a SEM JSM-5900 LV (JEOL, Tokyo, Japan), voltage 10–20 kV, pressure of 1E^−4^ Pa. The biological tissues were fixed with glutaraldehyde 2.5 wt.% in phosphate buffer 0.01 M for 1 hour. Finally, they were coated with a 5 nm gold layer. The energy dispersive analysis was done at 15 and 20 kV and the images were analyzed using Inca JEOL software. A carbon tape or fleshly cleaved mica on top of a SEM sample holder was used to support the samples.

#### Atomic force microscopy (AFM)

The sample’s topography was analyzed using an atomic force microscope (AFM) (Asylum Research, California, USA), operated in the tapping mode in air. Silicon probes (model Tap150Al-G, back side of the cantilever covered Al) with resonance frequencies of 150 kHz and force constant of 5 N/m were used. For the characterization of structure and roughness, height differences were evaluated.

### Sub-product identification

#### Thermogravimetric analysis (TGA)

TGA was performed using a Q500 (TA Instruments, USA). The samples (approx. 5 ± 0.1 mg) were collected in a standard platinum pan. The samples were dried in a vacuum oven at 70 °C to avoid degradation before being analyzed. The scan was run at 10 °C/min under a nitrogen flow. Mass change was measured from 40 °C to 800 °C. Each sample was analyzed three times.

#### Fourier transform infrared spectroscopy (FTIR)

FTIR spectra were recorded using a Nicolet 6700 spectrophotometer (Thermo Scientific, Massachusetts, USA) in a range of 500–4000 cm^−1^ and a resolution of 4 cm^−1^. The samples were dried in a vacuum oven at 70 °C to avoid degradation before being analyzed.

#### X-ray diffraction (XRD)

The XRD patterns were recorded in an Empyrean diffractometer using Cu Kα radiation in the range of 5–90° in steps of 1° for 24 s.

#### Solubility of the rosette like-microparticles

The solubility of the rosette-like microparticles after thermal treatment (heating between 25 °C to 1000 °C at 20 °C/min) were tested using the procedure described by Akbar *et al*. to determine reactive silica (SiO_2_) from fly ash^20^. Commercial silica and MWCNT were used as controls for silica and non-soluble carbon-based materials. Briefly, 5 mg of the microparticles were moistened and few drops of sulphuric acid were added. Next, 1 ml of hydrofluoric acid (HF) was added slowly and carefully (highly corrosive). The platinum container was placed in a water bath to evaporate the solution. Next, it was placed on a furnace and slowly heated to 1050 ± 10 °C. Finally, the residual material in the container was weighed to determine the soluble fraction that was not degraded. Also, a few drops of acetone were added to the platinum container and one drop of these suspensions was placed on a SEM grid to image the particles which remained. Similar solutions were placed in a plastic Eppendorf and the insoluble particles were precipitated and imaged by SEM.

## Results and Discussions

### Location of the microparticles in the pineapple peels

**Figure 1:**
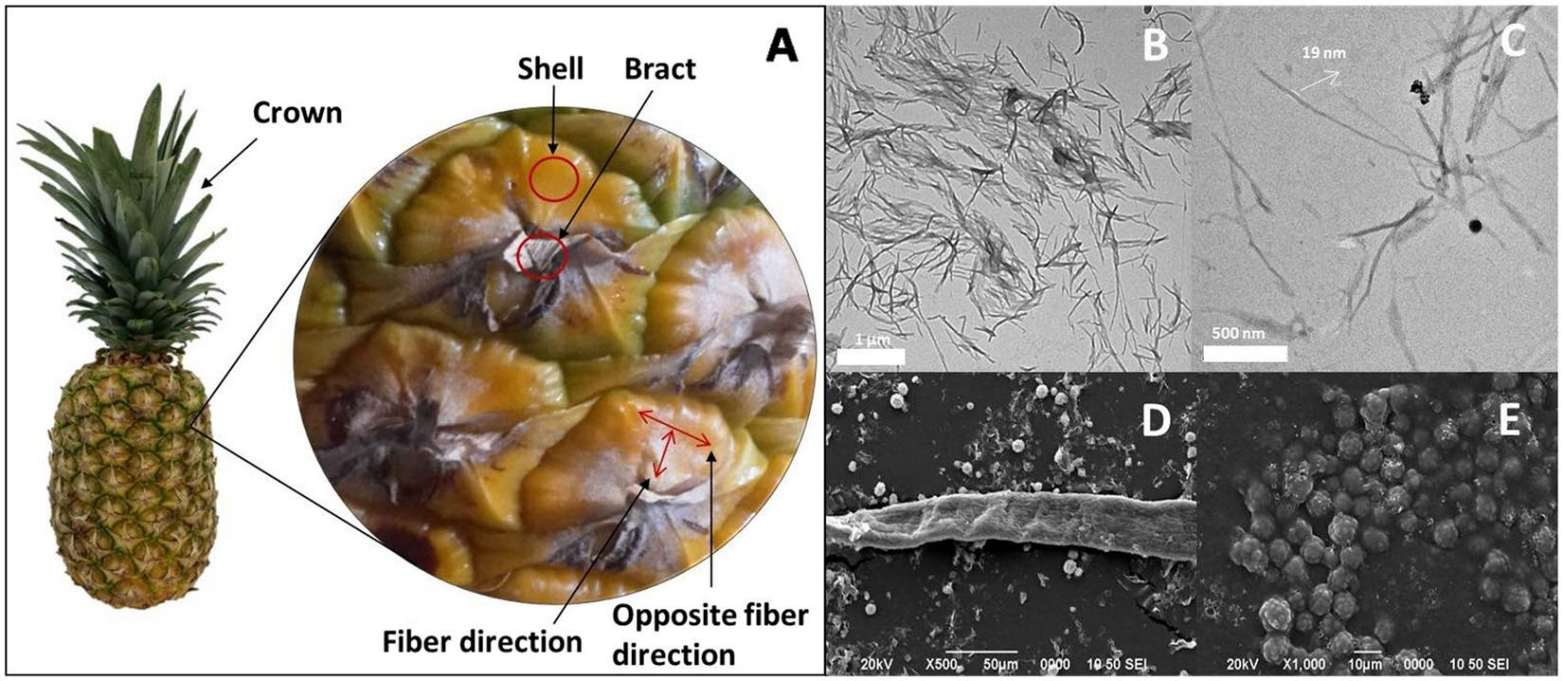
Pineapple parts, and TEM and SEM images of the nanocellulose and microparticles extracted. (**A**) Pineapple peel detail of parts investigated as sources of biogenic silica-based microparticles and nanocellulose. TEM images of microparticles present in the supernatants during the NCC extraction (after centrifugation at 2500 rpm and first incubation with H_2_SO_4_): (**B**) 3000X and (**C**) 10000X. SEM images of the precipitate after centrifugation: (**D**) 500X and (**E**) 1000X.

Microparticles with roundish morphology were previously described by Ferreira *et al*. as silica bodies present in the transversal sections of pineapple peels^7^. Microparticles forming a rosette-like assemble were found during the process of microcellulose extraction (Fig. 1). To further characterize and potentially develop a method for their extraction, the pineapple peels were imaged and analyzed.

**Figure 2:**
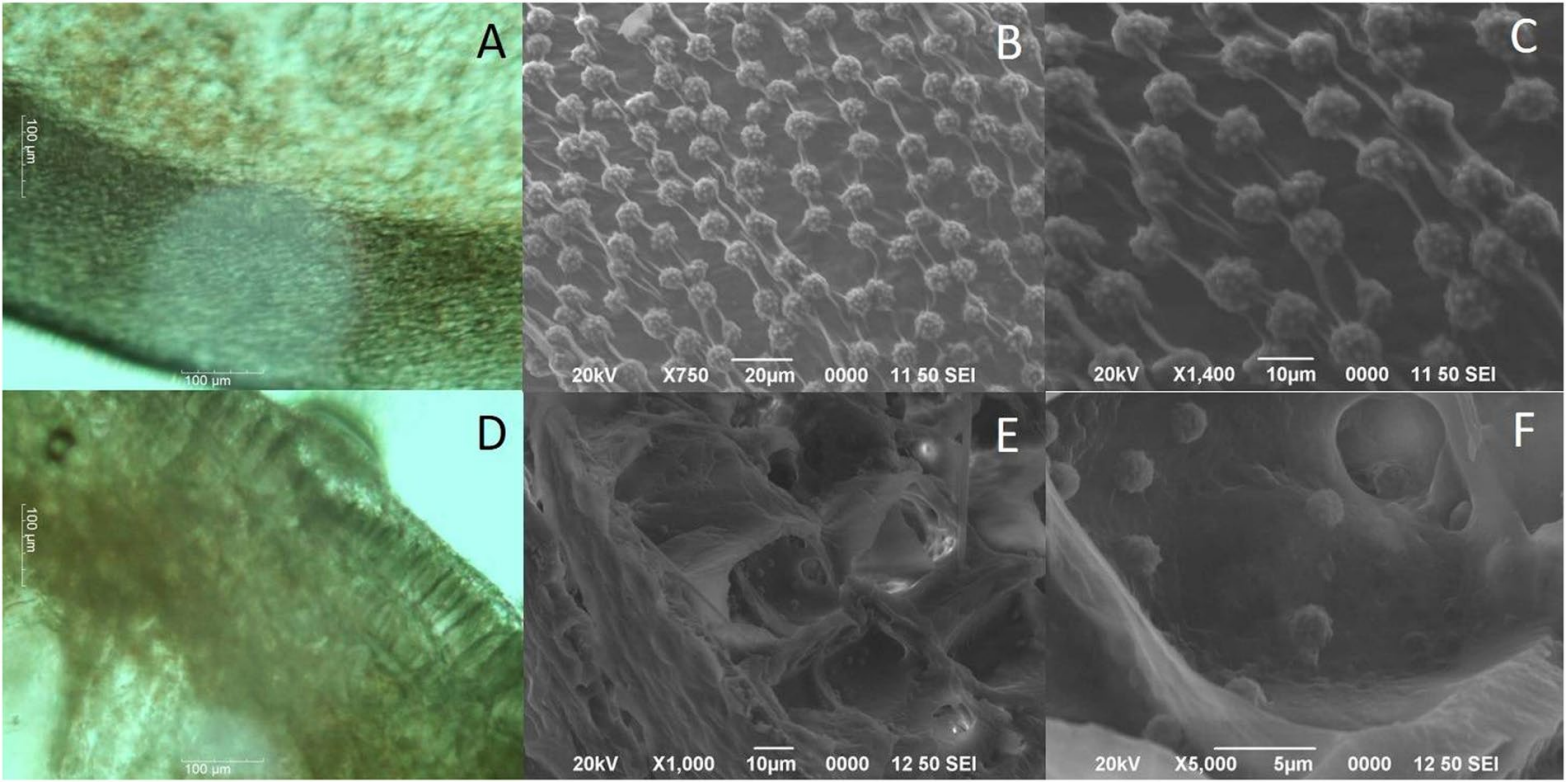
Microparticles present in specific plant tissues. Optical microscope images of the transverse section of the pineapple peel. (**A**) Opposite to the direction of fibers. (**D**) Along fiber direction. SEM images of the shell tissue section showed in A and D. SEM images of A (**B**) 750X and (**C**) 1400X. SEM images of D, (**E**) 1000X and (**F**) 5000X.

The rosette-like microparticles were absent from the crown tissue and the pulp (Supplementary Fig. S2). Only the bracts and the pineapple peel exocarp showed microparticles with similar sizes to the ones isolated during the NCC extraction process (Supplementary Fig. S2); interestingly, some of these particles were connected by some kind of fiber–like structure along one axis, suggesting an anisotropic pattern, (Fig. 2B,C) whereas in other cases the particles appeared to be loosely attached to the surface (Fig. 2E,F).

**Figure 3:**
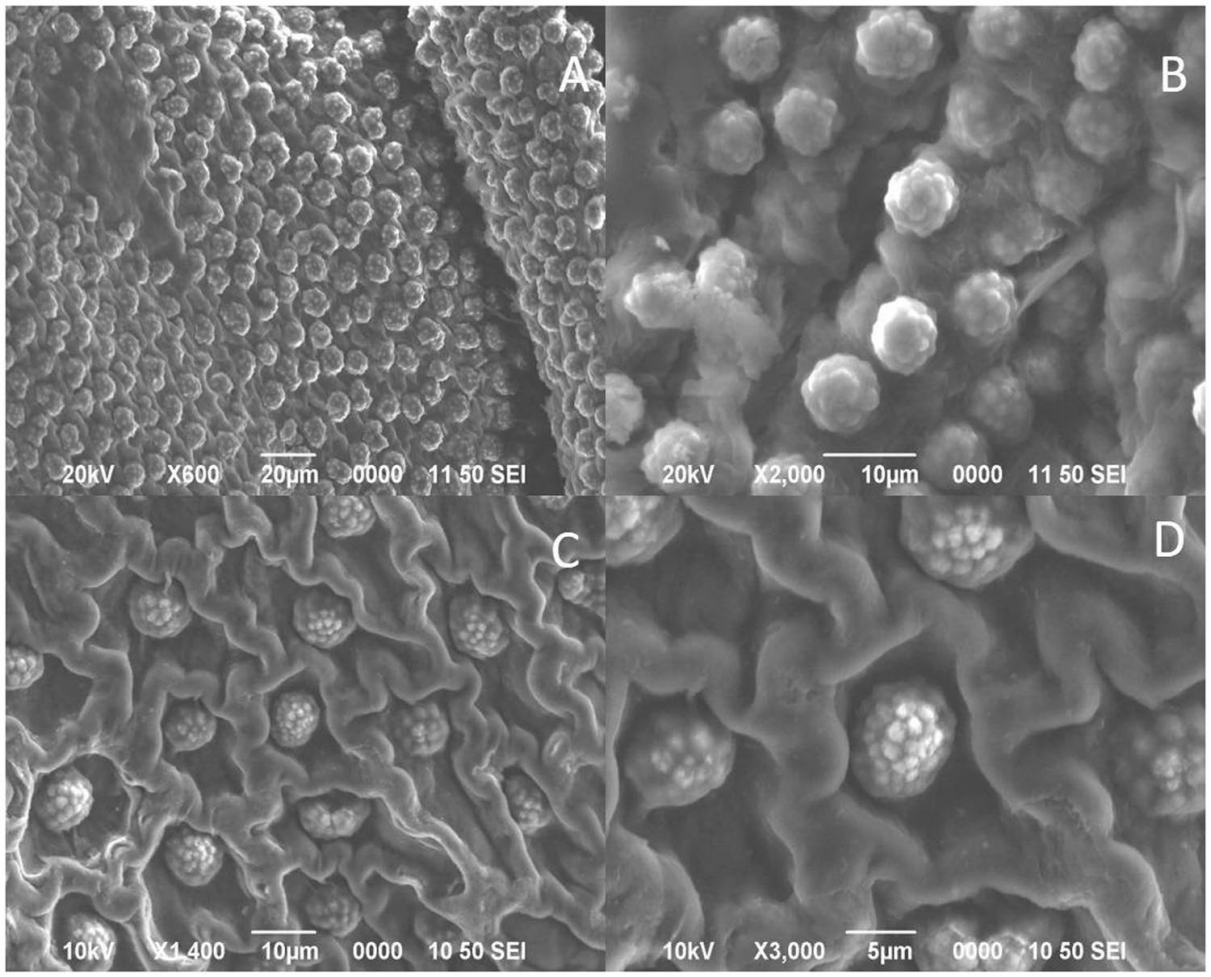
SEM images of the pineapple shell. In contact for 15 min with NaOH 20 wt.%: (**A**) 500X and (**B**) 2000X. In contact with H_2_SO_4_ 65 wt.%, 55 °C: (**C**) 1400X and (**D**)3000X.

To determine the nature of connecting fibers, segments of tissue were in contact with 20% NaOH or 65% H_2_SO_4_ at 55 °C for 15 min. Figure 3 suggests that the rosettes microparticles are bound preferentially between themselves by cellulose derivates, because the based structure was maintained, and the particles stayed interconnected after the NaOH treatment (Fig. 3A and B).

### Microparticles extraction process

**Figure 4:**
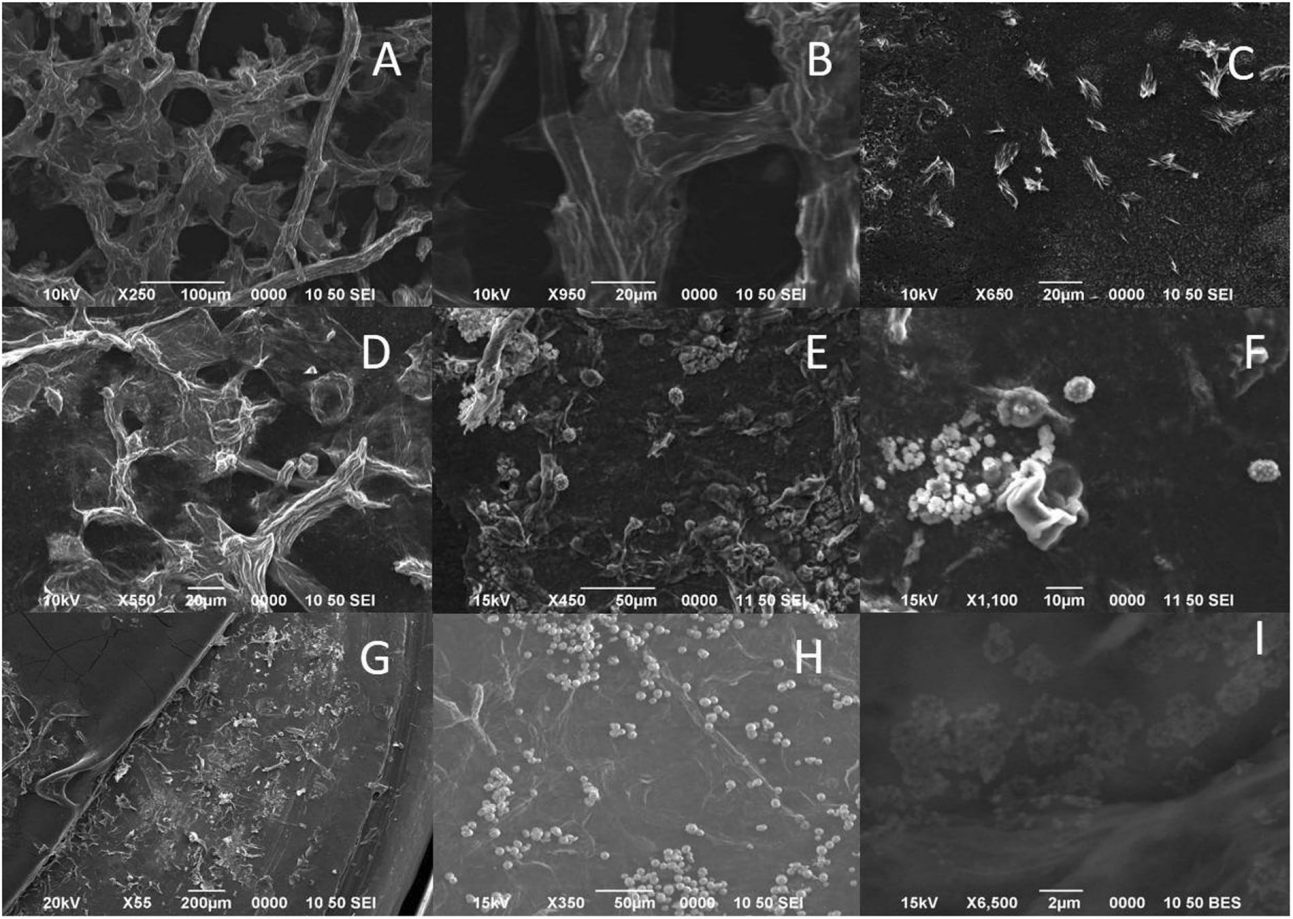
Solid after different treatments. Solid precipitated at 2500 rpm with different treatments after contact with the pineapple peels. (**A** and **B**) NaOH 20 wt.% and NaOH 12 wt.%. (**D** and **E**) NaOCl. (**G** and **H**) HCl. Solid remained in the supernatant after separating the fraction that precipitates at 2500 rpm with different treatments. (**C**) NaOH 20 wt.%. (**F**) NaOCl. (**I**) HCl.

To determine at which step of the NCC extraction the BRSM particles could be enriched, a sample of the solids that remained in the supernatants after each extraction step was analyzed by SEM. The BRSM particles bound by fibers can be imaged in the solid material after the NaOH treatment (Fig. 4A and B), and mainly smaller particles and fiber-like material were found in the supernatant (Fig. 4C).

After the NaOCl treatment, roundish silica nanoparticles could be imaged in small quantities (Fig. 4D and E). After the HCl step, a significant quantity of BRSM particles was imaged (Fig. 4G and H) but lower than in the precipitate centrifuged at 2500 rpm after incubation with H_2_SO_4_; suggesting that the BRSM separation is achieved mainly during the incubations with HCl and H_2_SO_4_. To verify that the microparticles do not remain in the supernatant during the last centrifugation step at 2500 rpm, a drop of the supernatant was dried in a SEM sample holder and imaged. Mainly, nanofibers with 23 ± 6 nm could be imaged (Supplementary Fig. S3). Interestingly, “pancake”-like clusters with similar diameter to the BRSM structures were found in the solid and supernatant of the HCl step (see Figs 4I and S4). These structures have similar partial elemental composition to the rosette-like microparticles according to the EDX partial elemental profile analysis (see below); suggesting that they could be the building blocks of the rosette-like structures. The results show that the microparticles can be extracted and separated in the HCl and H_2_SO_4_; being more abundant in the H_2_SO_4_ step.

To determine if the fiber-like structures bounding the BRSM are silica or cellulose, an EDX analysis of a single rosette-like microparticle that preserved the fiber-like structures was analyzed (Supplementary Fig. S5). No silicon was detected in the lateral fiber-like structures bound to the BRSM. This result suggests that the particles are bound to the extracellular matrix by cellulose-derived materials and not silicon dioxide derivates.

### Purification of the BRSM from cutins and nanocellulose

**Figure 5:**
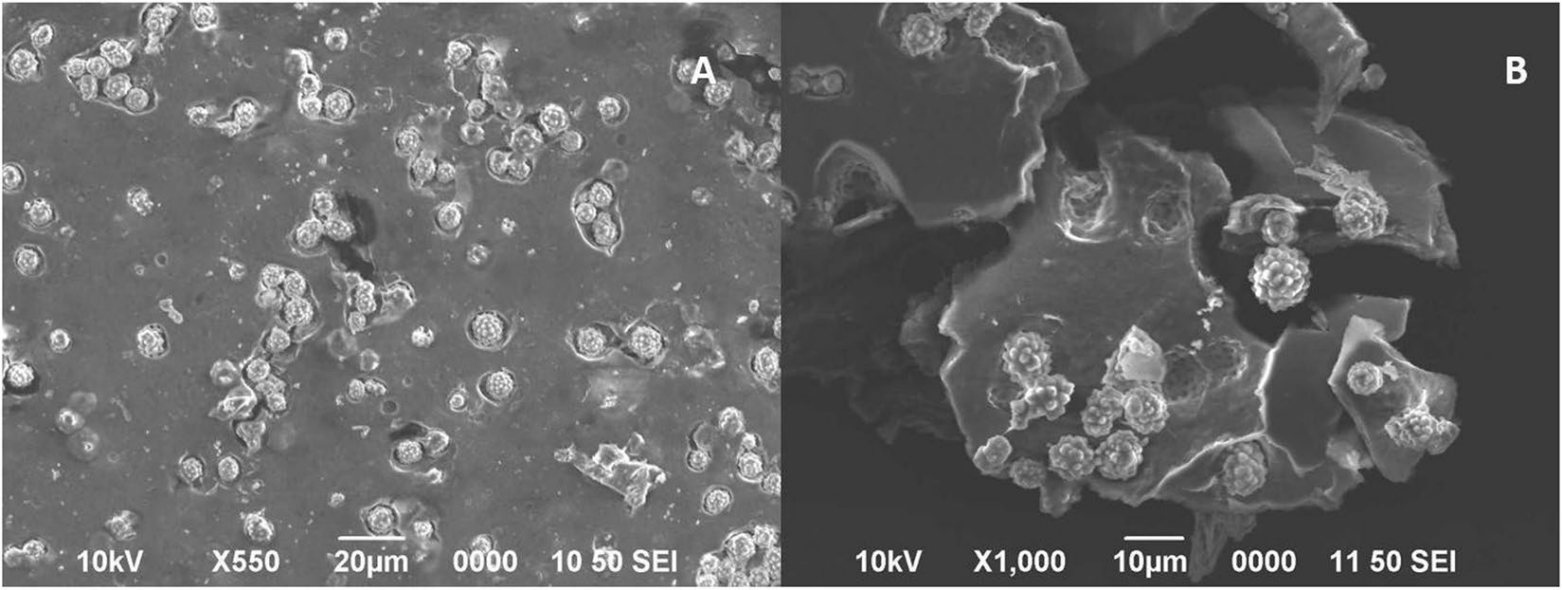
SEM images of the dried material precipitated from the H_2_SO_4_ solution at 2500 rpm. The material was washed three times with water before drying. (**A**) 550X, (**B**) 1000X.

**Figure 6:**
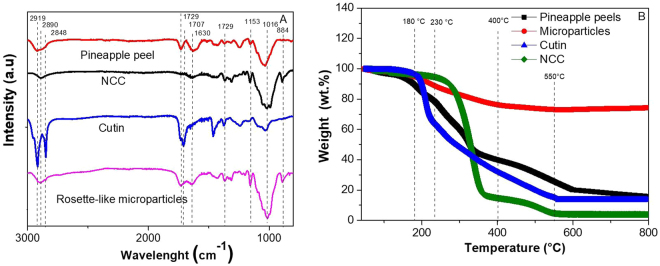
Comparative graph of pineapple peel, NCC, cutin and BRSM. (**A**) FTIR spectra, (**B**) TGA thermogram.

We then focus on the solid precipitated obtained after the first acid treatment, which contains high amounts of BRSM microparticles but also a resin that is formed by non-fibrillary structures (Fig. 5). The resin is associated to cutin (Fig. 6). The cutin derivate is an aliphatic biopolyester synthetized by epidermal cells that covers aerial plant surfaces. It functions as a waterproof barrier between plant tissues and the external environment. It is formed by a three-dimensional polymeric lipid composed of saturated and unsaturated fatty acid derivates^21^.

**Figure 7:**
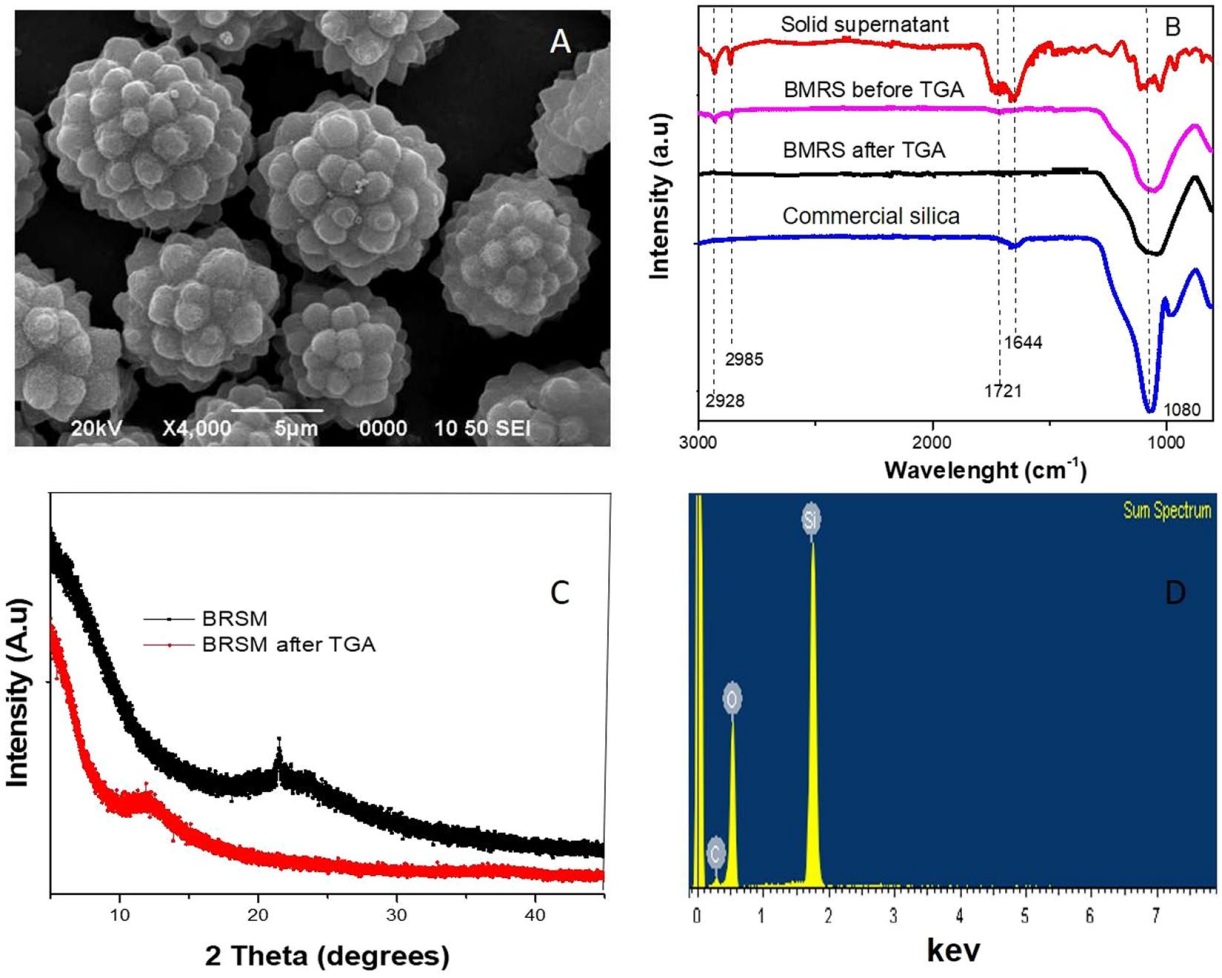
Characterization of microparticles obtained as a sub-product of the nanocellulose extraction process, (**A**) SEM image of the microparticles, (**B**) FTIR spectra of the solid in the supernatant separated after the second H_2_SO_4_step, (**C**) XRD spectra of BRSM before TGA and BRSM after TGA, (**D**) EDX spectra of BRSM.

The resin, however, does not have high affinity for the BRSM, favoring the separation process. The NCC and resin FTIR spectra comparison (Fig. 6) shows that the resin presents the two characteristic peaks of plant cutins at 2919 and 2848 cm^−1^, which are assigned to methylene asymmetric and symmetric stretching, respectively^22,23^. A peak at 1729 cm^−1^ is attributed to the stretching band vibration of esters. In contrast, there is only one peak at 2890 cm^−1^ associated with C-H stretching vibrations in the NCC^6^. Figure 7B shows the FTIR spectra of the material remained in suspension after the second H_2_SO_4_ incubation, showing peaks corresponding to cutins, along with remaining traces of nanocellulose, as showed by the peak at 884 cm^−1^. Therefore, using our extraction protocol BRSM can be purified along with traces of cutins and nanocellulose.

The spectra of the non-purified BRSM shows a very intense and broad band appearing between 890 cm^−1^ and 1200 cm^−1^ and centered at 1080 cm^−1^. In this region, the transversal optical (TO) and longitudinal optical (LO) modes of the Si-O-Si asymmetric stretching vibrations are assigned (Fig. 7B)^24^. However, peaks assigned to cutin and NCC are also detected.

The TGA analysis for pineapple peels, NCC, cutin derivates and microparticles showed that all samples readily lost weight step at 40–135 °C due to water evaporation (Fig. 6B and Supplementary Table S1). On one hand, pineapple peels contain cutin, lignin, hemicellulose and proteins, volatile compounds which are expected to degrade between 135 °C and 600 °C^6,25,26^; on the other hand, the silicon derivate compounds can be assigned to the 12.9 wt.% residue obtained from the pineapple peel according to the EDX, along with highly condensed aromatic structures formed by residual carbon^26^. A weight loss in the enriched BMRS solid fraction at temperatures lower than 450 °C was observed, which could occur due to the nanocellulose attached to the particles and other carbon based compounds, according to the EDX analysis. After the purification process more than 71 wt.% was not degraded at 800 °C; suggesting a high recovery of the silica.

### Characterization of the BRSM microparticles

**Figure 8:**
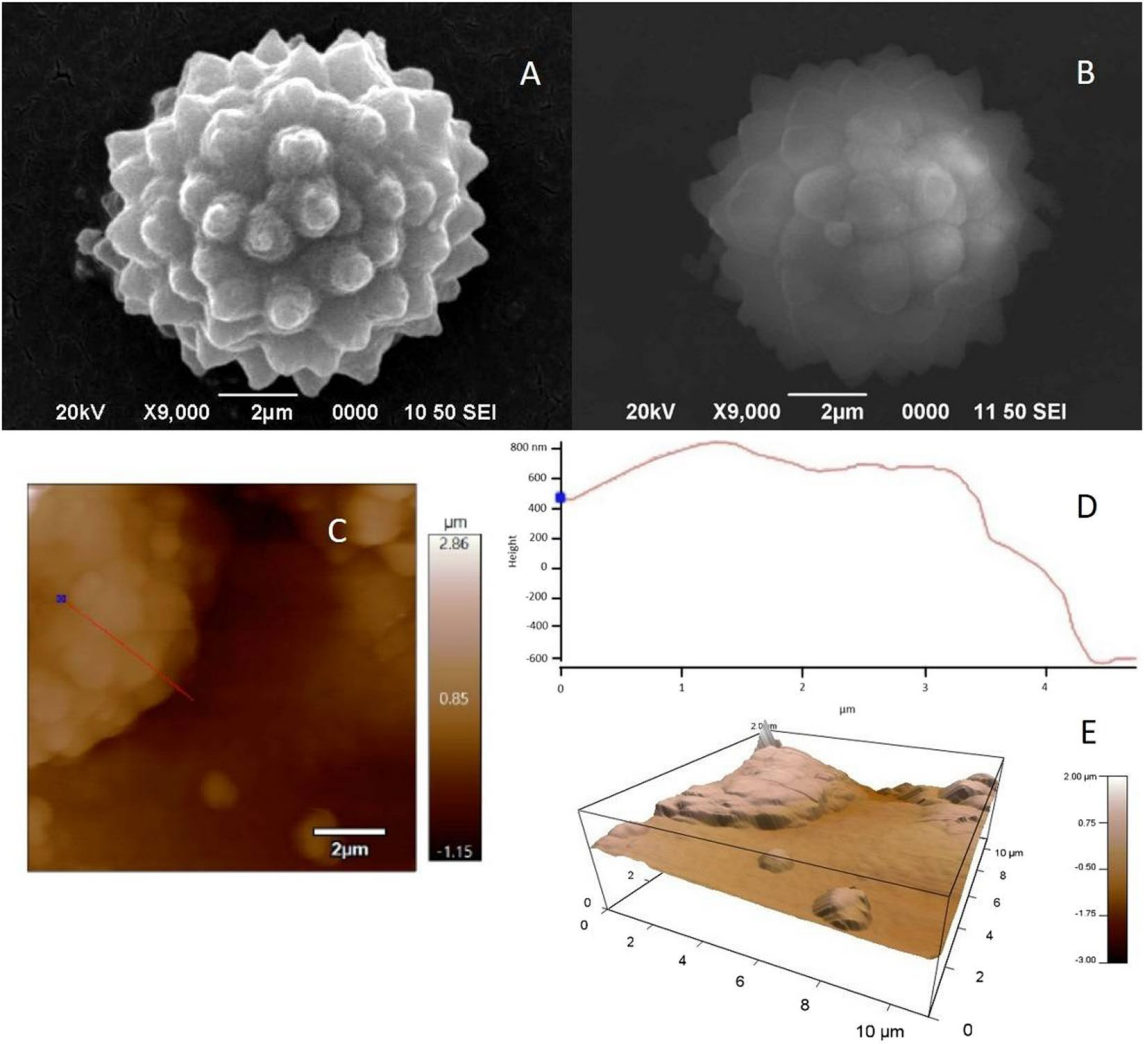
Microparticles and their regular nanometric features. SEM images of (**A**) BRSM before TGA, (**B**) BRSM after TGA, (**C**) AFM height image, (**D**) Cross-section of C, (**E**) 3D image of BRSM.

Rosette-like microparticles with an average size of 8.4 ± 2.5 μm were obtained before thermal treatment (Fig. 7A). FTIR spectra confirmed the presence of peaks associated to silicon dioxide (Fig. 7B). XRD analysis showed a broad band which could be related to amorphous silica and carbon amorphous materials (Fig. 7C)^27^. The peak at 22 °C is characteristic of crystalline cellulose. Other authors reported similar XRD spectra of silica particles coated with NCC^27^. EDX analysis of the purified samples showed that the particles are composed of carbon, oxygen and silicon (Fig. 7D). The average atomic concentration was: carbon 25.4 ± 5 at.%, oxygen 51.2 ± 2 at.% and silicon 20.5 ± 2 at.%. Finally, after thermal treatment, FTIR and XRD analysis showed the removal of crystalline NCC and cutin (Fig. 7B and C). Remarkably, up to 31 at.% of silicon was present in the final residues, and also the rosette-like structure was maintained (Fig. 8A and B).

AFM height images of single rosette-like structures show heights of approximately 750 nm for a rosette-like structure with 8 µm diameter (Fig. 8C). This suggests that the rosette-like structures could be formed by roundish silica nanoparticles that initially are built from the “pancake”-like layers as suggested in Supplementary Fig. S4. It is important to highlight that the microparticle size varies but their roundish nanometric features maintain a similar size; supporting the idea that these nanoparticles could be the building blocks of the BRSM. The particles extracted from pineapple peels from ripe fruits mainly contained micrometer size particles. Studies correlating the fruit growing stage could show the isolation of smaller particles.

To confirm that the BRSM particles are composed mainly of silica, they were treated with HF for 2 min. In general, silica particles are stable in the acid and basic solutions used before, but they could be dissolved in hydrofluoric acid^28^. Avicel, MWCNT and commercial silica were used as control materials^29,30^. The MWCNT were used to determine the solubility of carbon compounds that are not degraded at 1000 °C. The commercial silica particles and the BRSM were dissolved almost immediately in HF (as shown in Supplementary Fig. S6). The Avicel and MWCNT samples do not dissolve in contact with HF. In the case of the commercial silica and BRSM, after dissolving the particles and a thermal treatment at 1000 °C, no particles remained in the platinum containers. Our results confirm that the particles are mainly composed of silicon dioxide.

### Recovery yield of BRSM

The recovery yield of the nanocellulose and BRSM was calculated taken into account the following considerations: 10 wt.% of humid pineapple peel was turned into a dried solid and the 12.9 wt.% weight loss obtained from the TGA analysis of the pineapple peels was 100 % silica. The value of 6.45 g was set as maximum recovery mass. The experimental BRSM recovery of the process is 55.8%. More detailed information about the calculation is shown in Table 1.

**Table 1:** Biogenic rosette-like silica-based microparticles (BRSM) and nanocellulose (NCC) yield recovery.

Process (refer to Fig. S1)	Pineapple peels (A)	NaOH 20 wt.%, 1.5 h (B1)	NaOH 12 wt.%, 1 h (B2)	NaClO 2.5 wt.%, 2 h (C)	HCl 17 wt.%, 2 h (D)	H_2_SO_4_ 65 wt.%, 1.5 h (E)
**Nanocellulose extraction process**						
*Humid mass(g) (10 g humid peels: 1 g dried peels)	500	323	163	100	69	35
wt.% solid recovery (humid)	100%	64.6	32.6	20	13.8	7
**Separation and purification of BRSM and NCC**						
	**Mixture NCC and BRSM**	**Solid centrifugation (2500** **rpm)**	**NCC residual**			
^+^Mass (g) (dried)	5.5	3.6	1.9			
Solid recovery wt.% (dried)	100	65.5	34.5			
**Yield recovery of silica and NCC**						
**Calculation base 50 g of dried peels**				BRSM	NCC	
Experimental wt.% dried solid				7.2	3.8	

A, B1, B2, C, D and E: incubation with NaOH 20 wt.%, NaOH 12 wt.%, NaClO 2.5 wt.%, HCl 17 wt.% and H_2_SO_4_ 65 wt.% solutions, respectively.

*Humid mass weighted after rinsing with water until achieving stable pH. ^+^Mass obtained after rinsing with water until achieving a stable pH and drying at 50 °C. Solid recovery: (solid weighted after contact with the solution or separation by centrifugation and rinsing)/initial mass * 100.

## Conclusions

We showed 5 to 10 µm size rosette-like silica-based microparticles formed by even smaller micro and nanoparticles composing the bracts and shell of the pineapple. These microparticles are bound to cellulose-derived material, probably providing support and mechanical resistance to the shell structure, among other yet unexplored functions. This study showed the extraction, purification and characterization of the rosette-like microparticles. Further analysis are necessary to test the potential application and properties. Due to their large surface/volume ratio, applications such as adsorption of heavy metals and biomedical use should be considered. Because of the strong acids used in our methods, more environmental friendly extraction protocols need to be developed. The production of a valuable sub-product, such as silica microparticles from the pineapple biomass, should encourage efforts to improve the practices that deal with biomass disposal.

## Electronic supplementary material

Supplementary information
