# Supplementary material for: Biogenic silica-based microparticles obtained as a sub-product of the nanocellulose extraction process from pineapple peels
Source: Sci Rep. 2018 Jul 10;8:10417. doi: 10.1038/s41598-018-28444-4 (PMC6039511; doi:10.1038/s41598-018-28444-4)
Supplement: Supplementary file 1 — Supplementary information [file 41598_2018_28444_MOESM1_ESM.pdf]

Biogenic silica-based microparticles obtained as a sub-product of the nanocellulose extraction process from pineapple peels.

Yendry R. Corrales-Ureña[1, *], Carlos Villalobos-Bermúdez[1], Reinaldo Pereira[1], Melissa Camacho[1], Eugenia Estrada[2], Orlando Argüello-Miranda[1], Jose R. Vega-Baudrit[1, 3].

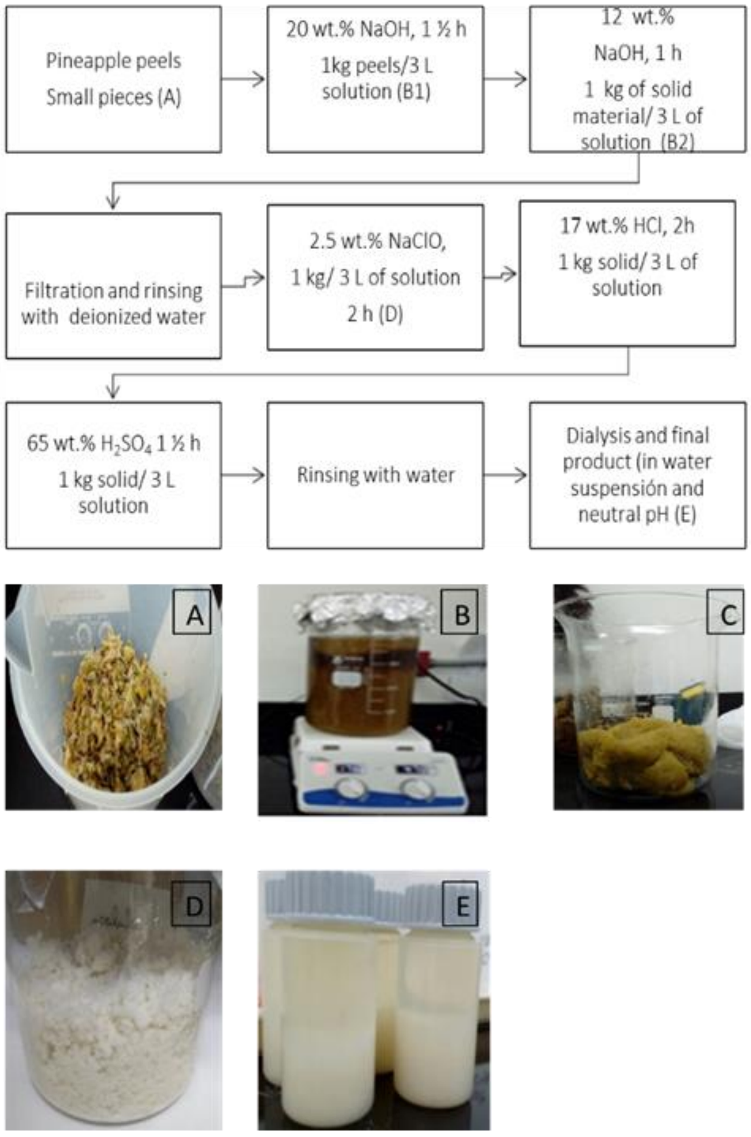

Supplementary Figure S1. Nanocellulose and rosette like silicon oxide based microparticles extraction diagram and illustrative images of the process.

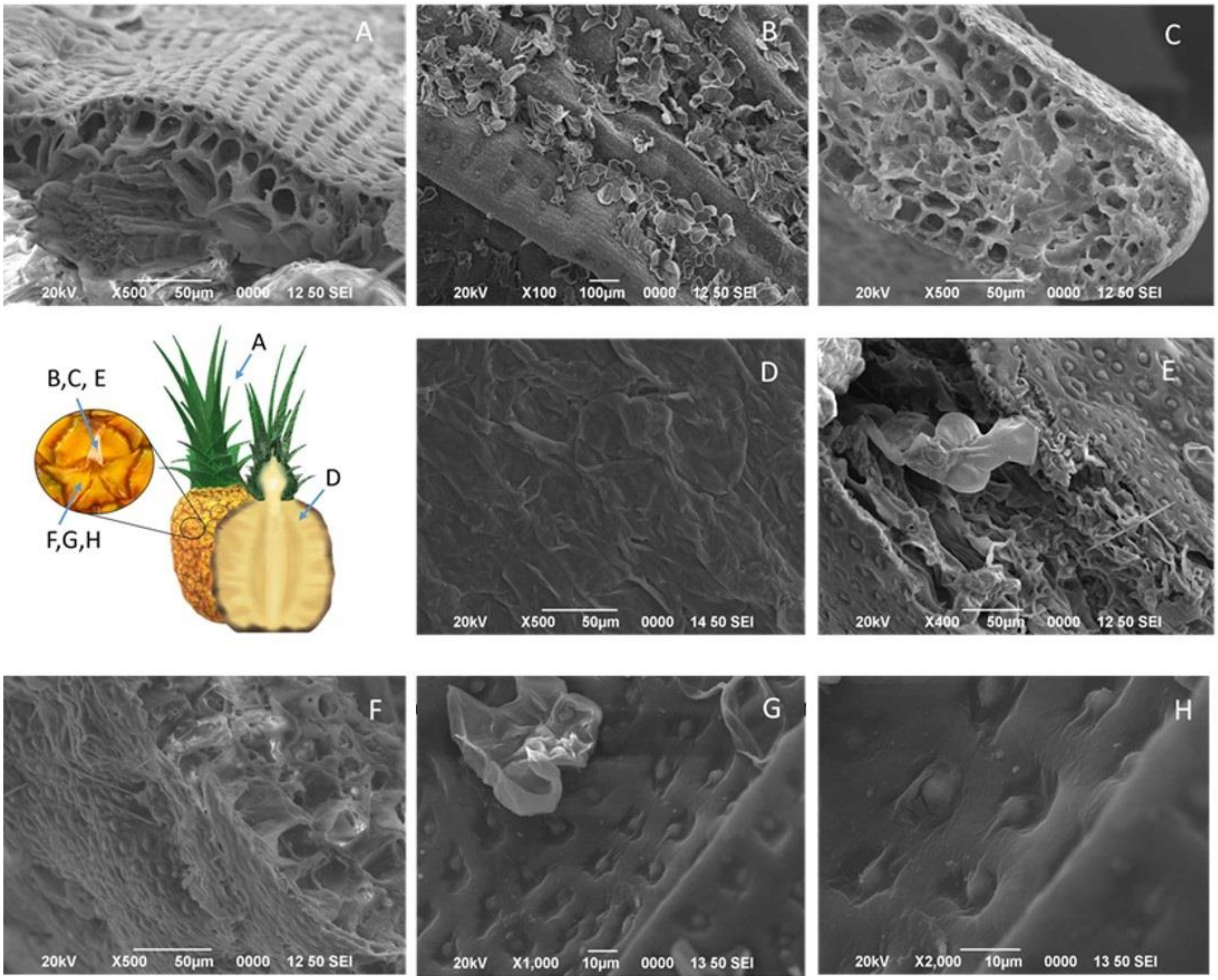

Supplementary Figure S2. SEM images of A) crown; B, C and E) bracts surface and transverse sections; D) pineapple pulp; F, G and H) pineapple shell.

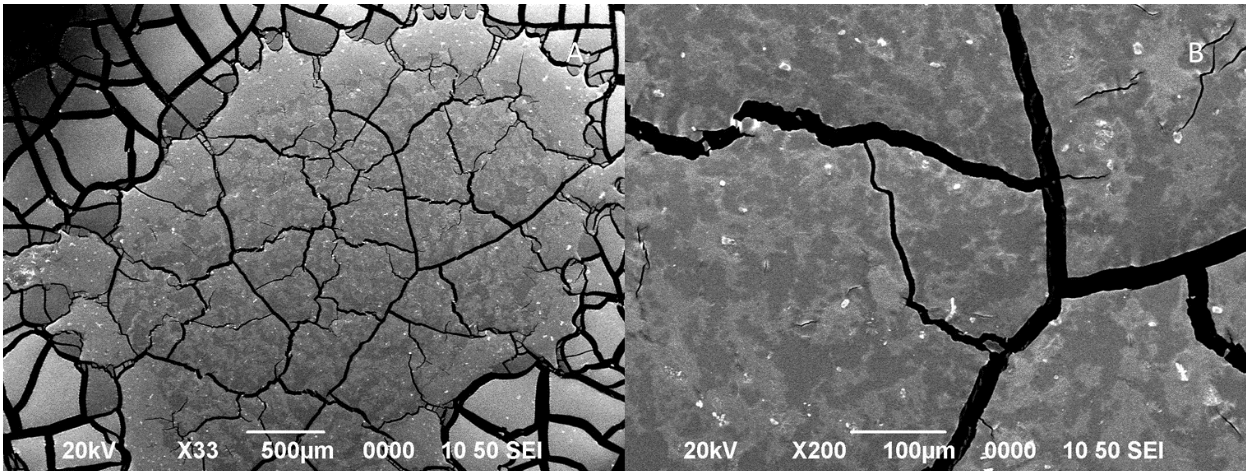

Supplementary Figure S3. SEM images of some drops of supernatant which remained after dispersing the final product in water and after separating the fraction that precipitates at 2500 rpm.

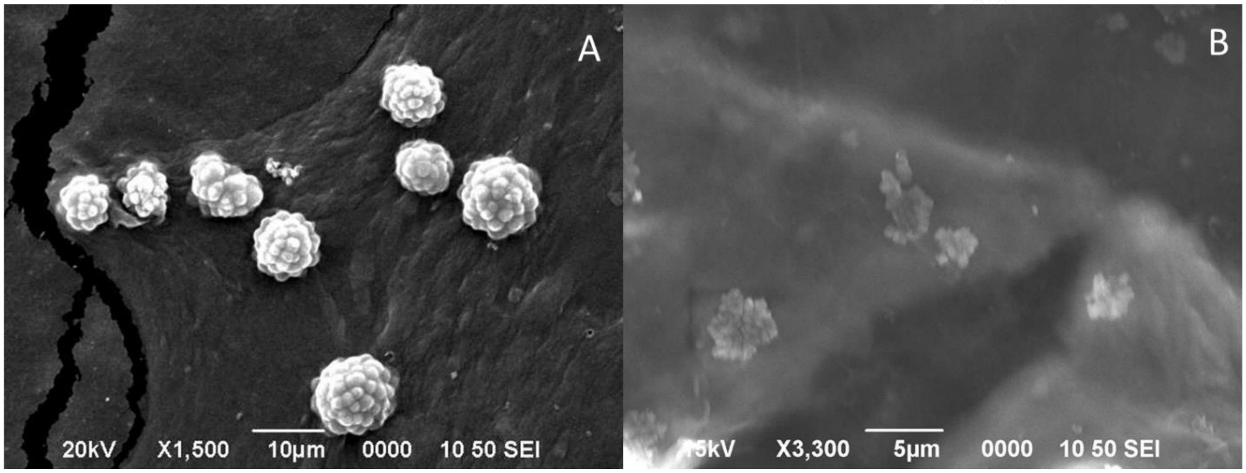

Supplementary Figure S4. SEM images of: A) 1500X rosette-like silica-based microparticles with different sizes but similar roundish silica building blocks B) 3300X pancake-like structures.

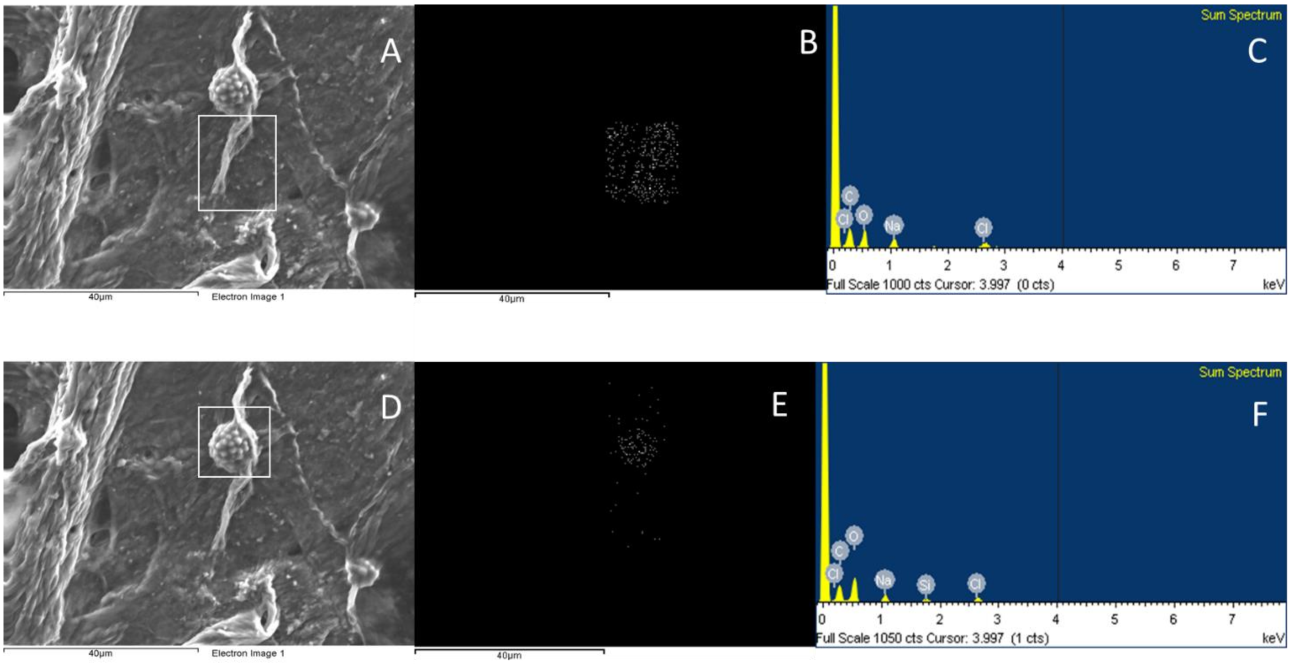

Supplementary Figure S5. Regions of the rosette like particles analyzed and EDX spectra.

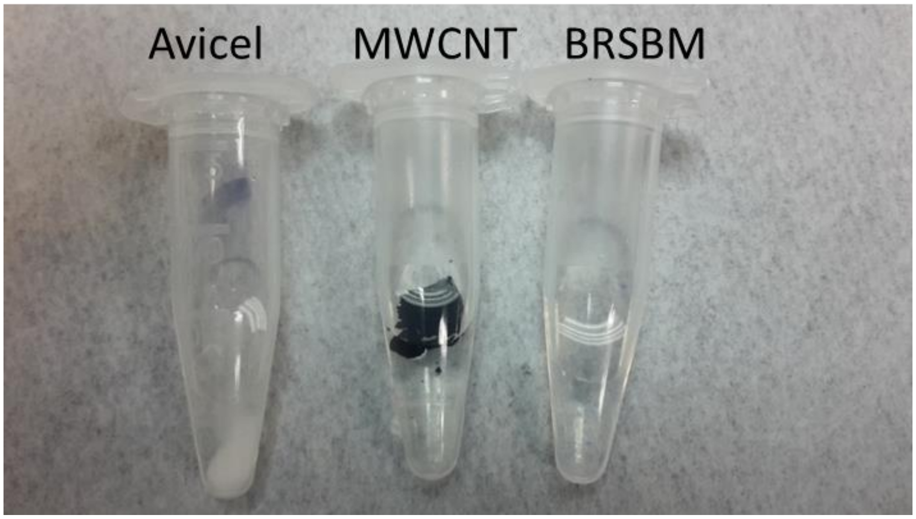

Supplementary Figure S6. Suspension of Avicel, MWCNT and BMBRS in HF 50 % after centrifugation at 13000 rpm.

Supplementary Table S1. Weight loss depending on the temperature analyzed by TGA of the pineapple peels, rosette like silicon oxide based microparticles and nanocellulose.

**Temperature (°C)**	**40-130**	**130-216**	**216-285**	**286-412**	**412-600**	**600-800**	**Residue**
Pineapple (wt.%)	3.8	14.8	18	25	19.5	4	12.9
**Temperature range**	**40-134**	**134-300**	**300-450**		**450-560**	**560-800**	
BRSBM (wt.%)	3	15	6		4	1.3	70.7
Nanocellulose (wt.%)	2.8	78.5			10.2	2	8.5
